# Inter­calated brucite-type layered cobalt(II) hydroxy­sulfate

**DOI:** 10.1107/S160053680902251X

**Published:** 2009-06-20

**Authors:** Bunlawee Yotnoi, Sanchai Luachan, Timothy J. Prior, Apinpus Rujiwatra

**Affiliations:** a Department of Chemistry, Faculty of Science, Chiang Mai University, Chiang Mai 50200, Thailand; b Department of Chemistry, University of Hull, Kingston upon Hull HU6 7RX, England

## Abstract

In an attempt to synthesize new cobalt(II) sulfate framework structures using 1,4-diaza­bicyclo­[2.2.2]octane as a template, crystals of poly[0.35-[hexa­aqua­cobalt(II)] [tri-μ-hydroxido-μ-sulfato-dicobalt(II)]], {[Co(H_2_O)_6_]_0.35_[Co_2_(OH)_3_(SO_4_)]}_*n*_, were obtained as a mixture with [Co(H_2_O)_6_]SO_4_ crystals. The crystal structure can be described as being constructed from discrete brucite-type [Co_4_(OH)_6_(SO_4_)_2_] layers, each of which is built up from edge-shared [Co(OH)_6_] and [Co(OH)_4_(OSO_3_)_2_] octa­hedra, with partial inter­calation by [Co(H_2_O)_6_]^2+^ ions. The absence of *ca* 30% of the [Co(H_2_O)_6_]^2+^ cations indicates partial oxidation of cobalt(II) to cobalt(III) within the layer.

## Related literature

The crystal structure of the title compound is closely related to that of Co_5_(OH)_6_(SO_4_)_2_(H_2_O)_4 _(Ben Salah *et al.*, 2004 ▶, 2006 ▶), which is composed of brucite-type cobalt hydroxide layers. The fundamental difference lies in the way that adjacent layers are linked; being pillared by ⋯O_3_SO—Co(H_2_O)_4_—OSO_3_⋯ groups in Co_5_(OH)_6_(SO_4_)_2_(H_2_O)_4_ but partially inter­calated by [Co(H_2_O)_6_]^2+^ ions in the title compound. For the crystal structures of layered materials of this type, see: Poudret *et al.* (2008 ▶). For a description of the Cambridge Structural Database, see: Allen (2002 ▶). 
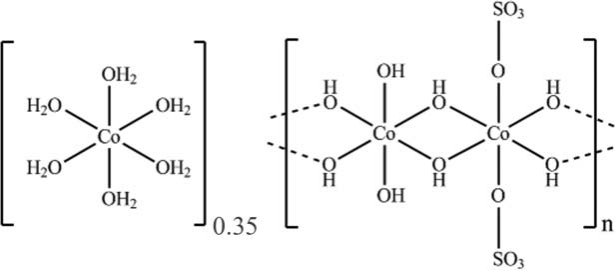


## Experimental

### 

#### Crystal data

[Co(H_2_O)_6_]_0.35_[Co_2_(OH)_3_(SO_4_)]
*M* *_r_* = 323.41
Trigonal, 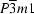

*a* = 6.3627 (19) Å
*c* = 12.180 (4) Å
*V* = 427.0 (2) Å^3^
*Z* = 2
Mo *K*α radiation
μ = 4.80 mm^−1^
*T* = 150 K
0.21 × 0.13 × 0.03 mm

#### Data collection

Stoe IPDS2 diffractometer
Absorption correction: multi-scan (*X-RED*; Stoe & Cie, 2008 ▶) *T*_min_ = 0.415, *T*_max_ = 0.862
1663 measured reflections
497 independent reflections
325 reflections with *I* > 2σ(*I*)
*R*_int_ = 0.097

#### Refinement

*R*[*F* ^2^ > 2σ(*F* ^2^)] = 0.048
*wR*(*F* ^2^) = 0.132
*S* = 0.90
497 reflections
40 parameters
3 restraints
Only H-atom coordinates refined
Δρ_max_ = 1.04 e Å^−3^
Δρ_min_ = −1.05 e Å^−3^

### 

Data collection: *X-AREA* (Stoe & Cie, 2008 ▶); cell refinement: *X-AREA*; data reduction: *X-RED* (Stoe & Cie, 2008 ▶); program(s) used to solve structure: *SHELXS86* (Sheldrick, 2008 ▶); program(s) used to refine structure: *SHELXL97* (Sheldrick, 2008 ▶); molecular graphics: *DIAMOND* (Brandenburg & Putz, 1999 ▶); software used to prepare material for publication: *PLATON* (Spek, 2009 ▶).

## Supplementary Material

Crystal structure: contains datablocks I, global. DOI: 10.1107/S160053680902251X/lh2832sup1.cif

Structure factors: contains datablocks I. DOI: 10.1107/S160053680902251X/lh2832Isup2.hkl

Additional supplementary materials:  crystallographic information; 3D view; checkCIF report

## Figures and Tables

**Fig. 1.**
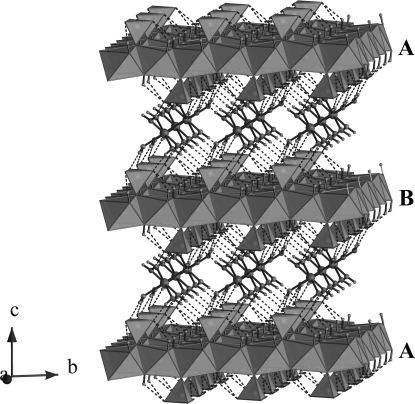
View of the crystal structure of I along the a axis showing the brucite-type [Co4(SO4)2(OH)6] layers which are intercalatated by the hydrogen bonded (dash lines) [Co(H2O)6]2+ ions.

**Fig. 2.**
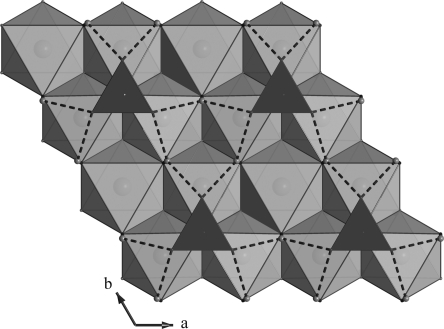
Polyhedral representation of the cobalt hydroxysulate layer, built up of the edge-shared [Co(OH)6] and [Co(OH)4(OSO3)2] octahedra.

**Fig. 3.**
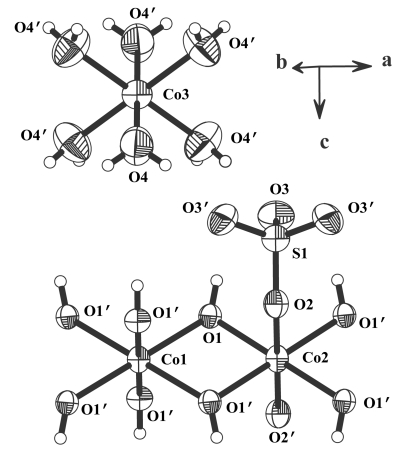
View of the extended asymmetric unit of I with atom numbering scheme. Displacement ellipsoids are drawn at the 80% probability level.

## supplementary crystallographic information

### Comment

Layered transition-metal hydroxides, in particular those exhibiting the brucite structure, have gained serious interest in both chemical and physical aspects. While the intercalation chemistry and potential application as catalysts are of the prime interest for the chemists, the physical interest primarily stems from the long-rang magnetic ordering in two dimensions. Examples of two dimensional triangular networks exhibiting such ordering are very rare, and the information on the crystal structures of layered materials of this type is yet rarer (Poudret, 2008). Thusfar there has been only one structure, Co_5_(OH)_6_(SO_4_)_2_(H_2_O)_4_, having the truely brucite-type magnetic layer (Ben Salah et al., 2004). The nuclear and magnetic structures, and magnetic properties of the compound were extensively reported (Ben Salah et al., 2006). The crystal structure of Co_5_(OH)_6_(SO_4_)_2_(H_2_O)_4_ consists of brucite-type cobalt hydroxide layers of edge-sharing octahedra, which are pillared by ··· O_3_SO—Co(H_2_O)_4_—OSO_3_··· groups. The compound exhibits ferromagnetic coupling with purely two dimensional magnetic ordering in an easy-plane magnet, which is a rare example of a single-layer magnet.

The crystal structure of [Co(H_2_O)_6_]_x_[Co_4_(SO_4_)_2_(OH)_6_] (I) where x ≈ 0.70 is closely related to that of Co_5_(OH)_6_(SO_4_)_2_(H_2_O)_4 _(Fig. 1), also comprised of brucite-type [Co_4_(SO_4_)_2_(OH)_6_] layers, each of which is built up from the edge-shared [Co(OH)_6_] and [Co(OH)_4_(OSO_3_)_2_] octahedra in the ab plane (Fig. 2). Two of three crystallographically distinct cobalt ions (Fig. 3), Co1 and Co2, are within the layers and located on the 1b and 3f Wyckoff sites, respectively. Unlike the Co_5_(OH)_6_(SO_4_)_2_(H_2_O)_4_ structure, the sulfate anion acts as a monodentate ligand and is coordinate covalently connected to the layered Co2 ion via the apical O atom, leaving the three basal O atoms pointing into the interlayered space (Fig. 3). The [Co_4_(SO_4_)_2_(OH)_6_] layers are stacked in the ABAB fashion along c, with a repeat distance of 12.180 (4) Å. In the interlayered gallery, there are intercalated discrete [Co(H_2_O)_6_]^2+^ ions in which the Co3 is located on the 1a site. These [Co(H_2_O)_6_]^2+^ ions are aligned in a way to maximize the hydrogen bonding interactions of O—H···O type between the aquo ligands and the basal O atoms of the layered sulfate pendants (Fig. 1). The orientation of the sulfate is also regulated by the hydrogen bonds established between the sulfate basal O atoms and the layered hydroxy groups. The Co—O bond length (2.065 (8) Å) within the hexaaquo ion is in good agreement with similar cations in the Cambridge Structural Database (Allen, 2002). The mean of similar Co—O distances is 2.09 (3) Å, but is notable that the majority of these structures were collected at room temperature. 97% of the structures containing cobalt hexaaquo ions are explicitly recorded at Co^2+^, none are recorded as Co^3+^.

The refined site occupancy of the intercalated [Co(H_2_O)_6_]^2+^ ions and the assumption of total charge neutrality imply the partial oxidation (15%) of the layer Co^II^ to Co^III^. This yields an overall composition [Co^II^(H_2_O)_6_]_0.7_[Co^II^_3.4_Co^III^_0.6_(SO_4_)_2_(OH)_6_] for I .

### Experimental

In the attempt to synthesize new cobalt(II) sulfate frameworks, crystals of I were unintentionally obtained from the hydrothermal reaction of cobalt(II) sulfate heptahydrate and 1,4-diazabicyclo[2.2.2]octane in acidic aqueous solution (pH 4.4) under autogenous pressure at 453 K for 72 h.

### Refinement

Hydrogen atoms were located by difference Fourier methods. The positions of these were refined subject to weak bond length restraints. Displacement parameters for the hydrogen atoms were set at 1.5 times the isotropic diaplcement parameter of the oxygen atom.

Prior to the refinement of site ocupancy of [Co(H_2_O)_6_]^2+^, all atoms were located using Fourier difference methods. The displacement parameters of the intercalating ion were anomalously large. There were large maxima and minima in the residual electron density: e-max = 2.59 e Å^-3^ (centered on Co2); e-min = -2.58 e Å^-3^. At this stage *wR*(F^2^) = 0.1914.

Refinement of the occupancy of the [Co(H_2_O)_6_]^2+^ cation resulted in a significant improvement in the quality of the fit to the data: e-max = 1.044 e Å^-3^; e-min = -1.046 e Å^-3 ^and *wR*(F^2^) = 0.132.

Careful inspection of the diffraction images did not reveal any weak reflections which might indicate ordering of the partially occupied cation.

### Crystal data

**Table:**

[Co(H_2_O)_6_]_0.35_[Co_2_(OH)_3_(SO_4_)]	*D*_x_ = 2.515 Mg m^−^^3^
*M**_r_* = 323.41	Mo *K*α radiation, λ = 0.71073 Å
Trigonal, *P*3*m*1	Cell parameters from 1764 reflections
Hall symbol: -P 3 2"	θ = 1.7–29.3°
*a* = 6.3627 (19) Å	µ = 4.80 mm^−^^1^
*c* = 12.180 (4) Å	*T* = 150 K
*V* = 427.0 (2) Å^3^	Plate, pale pink
*Z* = 2	0.21 × 0.13 × 0.03 mm
*F*(000) = 318.9	

### Data collection

**Table:**

Stoe IPDS2 diffractometer	497 independent reflections
Radiation source: fine-focus sealed tube	325 reflections with *I* > 2σ(*I*)
graphite	*R*_int_ = 0.097
Detector resolution: 6.67 pixels mm^-1^	θ_max_ = 29.3°, θ_min_ = 1.7°
ω scans	*h* = −7→8
Absorption correction: multi-scan (*X-RED*; Stoe & Cie, 2008)	*k* = −7→8
*T*_min_ = 0.415, *T*_max_ = 0.862	*l* = −14→16
1663 measured reflections	

### Refinement

**Table:**

Refinement on *F*^2^	Primary atom site location: structure-invariant direct methods
Least-squares matrix: full	Secondary atom site location: difference Fourier map
*R*[*F*^2^ > 2σ(*F*^2^)] = 0.048	Hydrogen site location: difference Fourier map
*wR*(*F*^2^) = 0.132	Only H-atom coordinates refined
*S* = 0.90	*w* = 1/[σ^2^(*F*_o_^2^) + (0.0839*P*)^2^] where *P* = (*F*_o_^2^ + 2*F*_c_^2^)/3
497 reflections	(Δ/σ)_max_ < 0.001
40 parameters	Δρ_max_ = 1.04 e Å^−^^3^
3 restraints	Δρ_min_ = −1.05 e Å^−^^3^

### Special details

**Table:**

Geometry. All e.s.d.'s (except the e.s.d. in the dihedral angle between two l.s. planes) are estimated using the full covariance matrix. The cell e.s.d.'s are taken into account individually in the estimation of e.s.d.'s in distances, angles and torsion angles; correlations between e.s.d.'s in cell parameters are only used when they are defined by crystal symmetry. An approximate (isotropic) treatment of cell e.s.d.'s is used for estimating e.s.d.'s involving l.s. planes.
Refinement. Refinement of *F*^2^ against ALL reflections. The weighted *R*-factor *wR* and goodness of fit *S* are based on *F*^2^, conventional *R*-factors *R* are based on *F*, with *F* set to zero for negative *F*^2^. The threshold expression of *F*^2^ > σ(*F*^2^) is used only for calculating *R*-factors(gt) *etc*. and is not relevant to the choice of reflections for refinement. *R*-factors based on *F*^2^ are statistically about twice as large as those based on *F*, and *R*- factors based on ALL data will be even larger.

### Fractional atomic coordinates and isotropic or equivalent isotropic displacement parameters (Å^2^)

**Table:**

	*x*	*y*	*z*	*U*_iso_*/*U*_eq_	Occ. (<1)
Co2	0.5000	0.0000	0.5000	0.0170 (3)	
Co1	0.0000	0.0000	0.5000	0.0163 (5)	
S1	0.6667	0.3333	0.2679 (2)	0.0214 (6)	
O1	0.1717 (3)	−0.1717 (3)	0.4215 (3)	0.0164 (9)	
O2	0.6667	0.3333	0.3913 (6)	0.0188 (15)	
O3	0.5403 (5)	0.0807 (9)	0.2298 (4)	0.0292 (11)	
Co3	0.0000	0.0000	0.0000	0.0250 (11)	0.700 (11)
O4	0.1574 (8)	0.3148 (16)	0.0920 (7)	0.039 (2)	0.700 (11)
H1	0.188 (9)	−0.188 (9)	0.355 (3)	0.059*	
H4	0.073 (9)	0.353 (18)	0.128 (5)	0.059*	0.700 (11)

### Atomic displacement parameters (Å^2^)

**Table:**

	*U*^11^	*U*^22^	*U*^33^	*U*^12^	*U*^13^	*U*^23^
Co2	0.0146 (5)	0.0156 (6)	0.0211 (6)	0.0078 (3)	0.0001 (2)	0.0002 (4)
Co1	0.0144 (6)	0.0144 (6)	0.0202 (9)	0.0072 (3)	0.000	0.000
S1	0.0220 (9)	0.0220 (9)	0.0203 (12)	0.0110 (4)	0.000	0.000
O1	0.0177 (16)	0.0177 (16)	0.0167 (18)	0.0111 (17)	−0.0002 (8)	0.0002 (8)
O2	0.017 (2)	0.017 (2)	0.023 (4)	0.0083 (11)	0.000	0.000
O3	0.034 (2)	0.023 (2)	0.027 (2)	0.0116 (12)	−0.0034 (10)	−0.0069 (19)
Co3	0.0256 (13)	0.0256 (13)	0.0238 (17)	0.0128 (7)	0.000	0.000
O4	0.034 (3)	0.043 (5)	0.044 (4)	0.022 (2)	−0.0080 (18)	−0.016 (4)

### Geometric parameters (Å, °)

**Table:**

Co2—O1	2.047 (3)	S1—O3^ix^	1.467 (5)
Co2—O1^i^	2.047 (3)	S1—O2	1.502 (7)
Co2—O1^ii^	2.047 (3)	O1—Co2^x^	2.047 (3)
Co2—O1^iii^	2.047 (3)	O1—H1	0.83 (3)
Co2—O2	2.264 (4)	O2—Co2^viii^	2.264 (4)
Co2—O2^i^	2.264 (4)	O2—Co2^ix^	2.264 (4)
Co1—O1^iv^	2.121 (4)	Co3—O4^xi^	2.065 (8)
Co1—O1^iii^	2.121 (4)	Co3—O4	2.065 (8)
Co1—O1^v^	2.121 (4)	Co3—O4^iv^	2.065 (8)
Co1—O1^vi^	2.121 (4)	Co3—O4^vi^	2.065 (8)
Co1—O1	2.121 (4)	Co3—O4^xii^	2.065 (8)
Co1—O1^vii^	2.121 (4)	Co3—O4^xiii^	2.065 (8)
S1—O3	1.467 (5)	O4—H4	0.82 (3)
S1—O3^viii^	1.467 (5)		
			
O1—Co2—O1^i^	180.000 (1)	O3^viii^—S1—O3^ix^	110.49 (19)
O1—Co2—O1^ii^	97.8 (2)	O3—S1—O2	108.4 (2)
O1^i^—Co2—O1^ii^	82.2 (2)	O3^viii^—S1—O2	108.4 (2)
O1—Co2—O1^iii^	82.2 (2)	O3^ix^—S1—O2	108.4 (2)
O1^i^—Co2—O1^iii^	97.8 (2)	Co2—O1—Co2^x^	102.00 (17)
O1^ii^—Co2—O1^iii^	180.0 (2)	Co2—O1—Co1	99.51 (14)
O1—Co2—O2	95.83 (13)	Co2^x^—O1—Co1	99.51 (14)
O1^i^—Co2—O2	84.17 (13)	Co2—O1—H1	112 (4)
O1^ii^—Co2—O2	95.83 (13)	Co2^x^—O1—H1	112 (4)
O1^iii^—Co2—O2	84.17 (13)	Co1—O1—H1	129 (7)
O1—Co2—O2^i^	84.17 (13)	S1—O2—Co2^viii^	125.79 (14)
O1^i^—Co2—O2^i^	95.83 (13)	S1—O2—Co2	125.79 (14)
O1^ii^—Co2—O2^i^	84.17 (13)	Co2^viii^—O2—Co2	89.3 (2)
O1^iii^—Co2—O2^i^	95.83 (13)	S1—O2—Co2^ix^	125.79 (14)
O2—Co2—O2^i^	180.0	Co2^viii^—O2—Co2^ix^	89.3 (2)
O1^iv^—Co1—O1^iii^	180.0 (2)	Co2—O2—Co2^ix^	89.3 (2)
O1^iv^—Co1—O1^v^	78.77 (13)	O4^xi^—Co3—O4	180.0 (4)
O1^iii^—Co1—O1^v^	101.23 (13)	O4^xi^—Co3—O4^iv^	86.7 (4)
O1^iv^—Co1—O1^vi^	101.23 (13)	O4—Co3—O4^iv^	93.3 (4)
O1^iii^—Co1—O1^vi^	78.77 (13)	O4^xi^—Co3—O4^vi^	86.7 (4)
O1^v^—Co1—O1^vi^	180.00 (15)	O4—Co3—O4^vi^	93.3 (4)
O1^iv^—Co1—O1	101.23 (13)	O4^iv^—Co3—O4^vi^	93.3 (4)
O1^iii^—Co1—O1	78.77 (13)	O4^xi^—Co3—O4^xii^	93.3 (4)
O1^v^—Co1—O1	78.77 (13)	O4—Co3—O4^xii^	86.7 (4)
O1^vi^—Co1—O1	101.23 (13)	O4^iv^—Co3—O4^xii^	86.7 (4)
O1^iv^—Co1—O1^vii^	78.77 (13)	O4^vi^—Co3—O4^xii^	180.0 (4)
O1^iii^—Co1—O1^vii^	101.23 (13)	O4^xi^—Co3—O4^xiii^	93.3 (4)
O1^v^—Co1—O1^vii^	101.23 (13)	O4—Co3—O4^xiii^	86.7 (4)
O1^vi^—Co1—O1^vii^	78.77 (13)	O4^iv^—Co3—O4^xiii^	180.0 (4)
O1—Co1—O1^vii^	180.0 (2)	O4^vi^—Co3—O4^xiii^	86.7 (4)
O3—S1—O3^viii^	110.49 (19)	O4^xii^—Co3—O4^xiii^	93.3 (4)
O3—S1—O3^ix^	110.49 (19)	Co3—O4—H4	120 (5)

Symmetry codes: (i) −*x*+1, −*y*, −*z*+1; (ii) −*x*+*y*+1, −*x*, *z*; (iii) *x*−*y*, *x*, −*z*+1; (iv) −*x*+*y*, −*x*, *z*; (v) *y*, −*x*+*y*, −*z*+1; (vi) −*y*, *x*−*y*, *z*; (vii) −*x*, −*y*, −*z*+1; (viii) −*y*+1, *x*−*y*, *z*; (ix) −*x*+*y*+1, −*x*+1, *z*; (x) −*y*, *x*−*y*−1, *z*; (xi) −*x*, −*y*, −*z*; (xii) *y*, −*x*+*y*, −*z*; (xiii) *x*−*y*, *x*, −*z*.

### Hydrogen-bond geometry (Å, °)

**Table:**

*D*—H···*A*	*D*—H	H···*A*	*D*···*A*	*D*—H···*A*
O1—H1···O3^x^	0.83 (3)	2.54 (5)	3.125 (6)	129 (4)
O1—H1···O3	0.83 (3)	2.54 (5)	3.125 (6)	129 (4)
O4—H4···O3^vi^	0.82 (3)	1.90 (9)	2.712 (1)	170 (7)

Symmetry codes: (x) −*y*, *x*−*y*−1, *z*; (vi) −*y*, *x*−*y*, *z*.
